# 3D reconstruction of coronary arteries from 2D angiographic projections using non-uniform rational basis splines (NURBS) for accurate modelling of coronary stenoses

**DOI:** 10.1371/journal.pone.0190650

**Published:** 2018-01-03

**Authors:** Francesca Galassi, Mohammad Alkhalil, Regent Lee, Philip Martindale, Rajesh K. Kharbanda, Keith M. Channon, Vicente Grau, Robin P. Choudhury

**Affiliations:** 1 Radcliffe Department of Medicine, Oxford Acute Vascular Imaging Centre, University of Oxford, Oxford, United Kingdom; 2 Radcliffe Department of Medicine, Division of Cardiovascular Medicine, University of Oxford, Oxford, United Kingdom; 3 Nuffield Department of Surgical Sciences, University of Oxford, Oxford, United Kingdom; 4 Oxford Heart Centre, NIHR Oxford Biomedical Research Centre, Oxford University Hospitals NHS Foundation Trust, Oxford, United Kingdom; 5 Department of Engineering Science, Institute of Biomedical Engineering, University of Oxford, Oxford, United Kingdom; Heart Research Institute, AUSTRALIA

## Abstract

**Objective:**

Assessment of coronary stenosis severity is crucial in clinical practice. This study proposes a novel method to generate 3D models of stenotic coronary arteries, directly from 2D coronary images, and suitable for immediate assessment of the stenosis severity.

**Methods:**

From multiple 2D X-ray coronary arteriogram projections, 2D vessels were extracted. A 3D centreline was reconstructed as intersection of surfaces from corresponding branches. Next, 3D luminal contours were generated in a two-step process: first, a Non-Uniform Rational B-Spline (NURBS) circular contour was designed and, second, its control points were adjusted to interpolate computed 3D boundary points. Finally, a 3D surface was generated as an interpolation across the control points of the contours and used in the analysis of the severity of a lesion. To evaluate the method, we compared 3D reconstructed lesions with Optical Coherence Tomography (OCT), an invasive imaging modality that enables high-resolution endoluminal visualization of lesion anatomy.

**Results:**

Validation was performed on routine clinical data. Analysis of paired cross-sectional area discrepancies indicated that the proposed method more closely represented OCT contours than conventional approaches in luminal surface reconstruction, with overall root-mean-square errors ranging from 0.213mm^2^ to 1.013mm^2^, and maximum error of 1.837mm^2^. Comparison of volume reduction due to a lesion with corresponding FFR measurement suggests that the method may help in estimating the physiological significance of a lesion.

**Conclusion:**

The algorithm accurately reconstructed 3D models of lesioned arteries and enabled quantitative assessment of stenoses. The proposed method has the potential to allow immediate analysis of the stenoses in clinical practice, thereby providing incremental diagnostic and prognostic information to guide treatments in real time and without the need for invasive techniques.

## Introduction

Coronary artery disease is characterized by narrowings, termed stenoses, that impair blood flow to the heart muscle. Two-dimensional invasive coronary X-ray arteriography (ICA) remains the best modality for the assessment of patients at intermediate to high-risk of this extremely common condition [1]. ICA has advantages of high temporal (up to 60 frames/sec) and spatial resolution (~0.3mm), so that even small coronary lumen alterations are captured. Furthermore, it is an interventional imaging modality, which not only provides diagnostic information but also allows for subsequent therapeutic procedures.

Nevertheless, an inherent limitation of ICA is that it consists of 2D projection images of a complex 3D anatomy, and is therefore prone to artifacts and inconsistencies due to vessel overlap and foreshortening. In addition, the number of views available is limited (in order to limit X-ray exposure and adverse effects of higher amounts of contrast agent). Moreover, interpreting the images and correlating information from the separate angiograms remains partially subjective. This can lead to inter-observer and intra-observer variability and, most importantly, to inaccurate estimation of lesion severity and incorrect selection of stent size [2]. To compensate the fundamental limitation of ICA and, hence, quantify accurately the lumen disease, in the last two decades, a variety of methods have been developed for the 3D reconstruction of coronary arteries from a limited number of 2D projections.

The most recent review about coronary artery reconstruction provides a comprehensive overview of the subject [3]. Among the various methods, back-projection methods are suitable for any type of X-ray angiography modalities (i.e. monoplane, biplane, rotational) and are designed to work with calibrated and non-calibrated data [4–8]. Generally, prior to 3D reconstruction, a minimum number of 2D point correspondences are defined (e.g. by visual inspection) and are used to estimate the optimal geometry that characterizes the position and orientation of one projection image relative to the other [4,5].

Correspondence establishment is an essential and critical step in back-projection methods, as the 3D vessel centreline points are reconstructed by triangulating the correspondences. A standard approach is to apply epipolar constraint [4–9]. Given a point in the first view, its corresponding point in the second view must lie on the generated epipolar line. However, isocentre offsets and mechanical distortions of the X-ray system together with small perspective projection angles can greatly deteriorate the epipolar constraint, leading to inaccurate correspondence. In addition, in most cases, the epipolar constraint does not yield a single match in the second view. One solution to this problem is to calculate the optimal mapping that minimizes the sum of distance errors from the epipolar line over the vessel centreline [8]. Another solution is to introduce soft epipolar constraints that expand the search for the matching point around the epipolar line [7,9]. In order to avoid the problem of point-to-point correspondences, a strategy based on branch-to-branch correspondences was recently proposed [10]. To this end, centreline branches were labelled on each projection exploiting the spatial coherence existing across the image sequence.

The approach presented in this paper to reconstruct 3D vessel centrelines has similar characteristics to the work presented by Cardenes et al. [10]. Similarly, it considers a centreline segment as a curve joining two nodes (bifurcation points) and it labels segments using the connectivity matrix of the coronary tree image. Additionally, in our method, the ends (nodes) of corresponding branches (segments) are employed as landmark points for estimating the optimal geometry parameters. Importantly, no assumption based on index correspondences is presumed, which can be inaccurate for perspective projections of a tortuous structure, and 3D centrelines of paired segments are reconstructed as intersection of two surfaces.

The reconstruction of the 3D vessel lumen is a crucial step for the assessment of stenosis severity. A common approach is to approximate vessels with tubular objects that are reconstructed by sweeping a circular cross-section along a spine (3D centreline) [4,10–12]. Diameter values are computed on a view as the Euclidean distance between two border points of the vessel centred at a centreline point and then corrected for geometric magnification. More accurate approaches account for the fact that, due to the rotational movements of the X-ray source, the projections used for the calculation are in general not perpendicular to the normal of the cross sectional planes of the centreline [5,8]. Some investigators use diameter information from two views to fit an ellipse to the cross section [13]. Other investigators use the diameter vectors from two views directly as the axes of an elliptic cross-section [14]. However, because of the lack of triple orthogonality, the ellipse can be ambiguous, and ambiguity increases with a decreasing angle between the two diameters. In addition, the non-orthogonality of contours to the vessel centreline usually yields irregularities in the 3D surface (i.e. contours intersecting each other).

Generally, the circular cross-section approximation simplifies and speeds up the reconstruction process, and it may be sufficiently accurate in the case of healthy vessels. However, when coronary arteries are diseased and may contain a significant extent of atherosclerotic plaque, their cross-section can change from roughly circular to various arbitrary shapes, depending on the extent and relative orientation of the plaque [15]. To address this issue, we propose a strategy that aims to model a luminal template cross-section to an extent that is consistent with the stenosis profile on each view, using Non-uniform Rational Basis Splines (NURBS) [16].

In order to provide validation for the approach, we compared lesion assessment using this novel 3D method with Optical Coherence Tomography (OCT), an invasive imaging modality that enables high-resolution endoluminal visualization of coronary *anatomy* [17]. Additionally, we investigated the correlation between the volume reduction due to a lesion and the Fractional Flow Reserve (FFR) measurement, an invasive technique that uses vessel *physiology* to gauge the functional significance of a stenotic lesion [18].

## Materials and methods

### Algorithm

**Fig 1:**
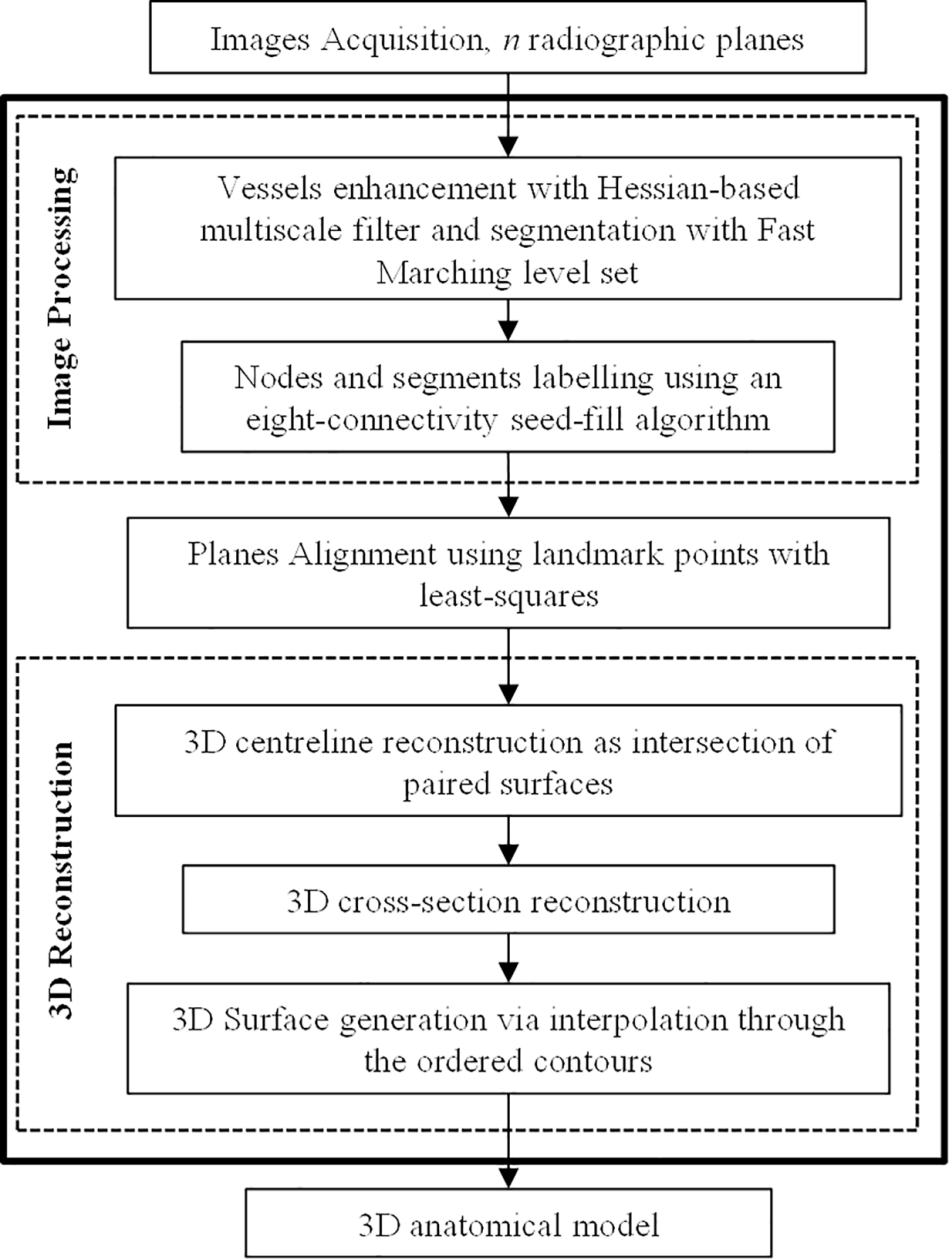
Schematic pipeline of the reconstruction algorithm.

The algorithm comprises two main steps: (1) 2D pre-processing of the projections to identify the stenotic vessel (in terms of centreline and profiles), and (2) 3D processing to obtain a 3D model of the vessel and stenotic segment (Fig 1). The main contribution of our work resides in the second step.

#### 2D projections processing: Image enhancement and vessel extraction

**Fig 2:**
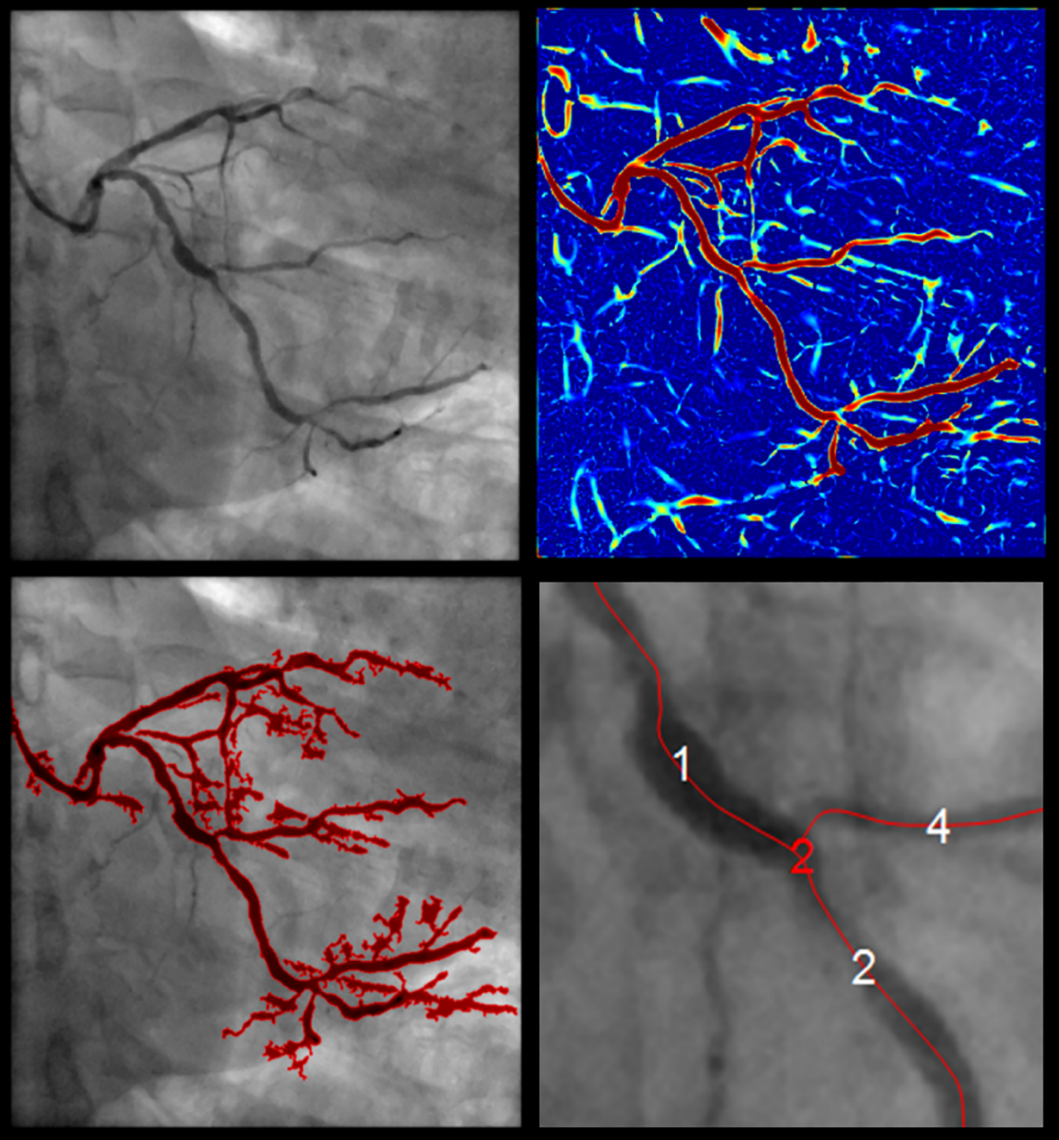
2D pre-processing. From the left: 2D angiographic image (pixel resolution of 512x512 and image pixel spacing of 0.278mm); vesselness image (red colour indicates higher response); extracted coronary tree; labelled segments (white) and nodes (red) overlaid the original image.

Images were firstly pre-processed using a classic Hessian-based multiscale filter, which detects tubular structures while removing background anatomy, such as bones and muscle tissues [19]. The filter was calculated for different scales in order to enhance vessels with different diameters (seven linearly increasing scales ranging between 0.5mm and 4mm, which approximately corresponded to the radius range of projected coronary arteries). The resulting vesselness image, i.e. the maximum response for each pixel, provided a measure for the probability of each pixel to belong to a vessel (Fig 2).

2D vessel boundaries were extracted using a fast marching level set method on the vesselness image, by interpreting vessel boundaries as the propagating front final position [20,21]. The front expanded outward from a seed point towards an end point selected by the user (by simple mouse clicking), and with speed inversely proportional to the gradient magnitude of the vesselness image i.e. within a vessel, the gradient is low, and the front propagates fast, while at the boundaries the gradient is high and the front slows down. A constrained map was defined such that the front propagation was constrained to values within the range 0.1–1 (a low boundary >0 was required to avoid losing regions that show low vessel-like structures due to insufficient contrast). The exact position of the selected initialization points did not influence the behaviour of the fast marching process, as long as they were located within a vessel. Selection of one start and one end points worked robustly for a targeted arterial segment. The user could select multiple initialization points, for detection of multiple vessels. The subsequent analysis proceeded automatically i.e. no additional manual interaction.

To compute the vessel centreline a subvoxel precise skeletonization method was applied [22]. The centreline was computed as the minimum-cost path from an end to point to a start point by performing a gradient descent on the fast marching distance map (cost function).

Given the skeleton of a coronary tree in the form of a binary image, then nodes (i.e. branching points) and segments (i.e. subsets of the skeleton that are bifurcation free) were detected and labelled on each projection by exploiting the connectivity matrix of the binary image. To this end, connected pixels were found using an eight-connectivity seed-fill algorithm [23]. The seed-fill algorithm started from a seed point (a foreground pixel) and then iteratively searched its neighbours to detect connected components. For different projections, labels could propagate differently. To ensure correspondence of labels, the initialization points previously selected for vessels extraction (i.e. fast marching seed points) were employed. These points were indicated so that they corresponded across projections. An initialization point was matched to a node based on the Euclidean distance. Only nodes having a matching initialization point were retained. Therefore, node labels across projections were ensured to correspond, and so were branches. Finally, the 2D centrelines were represented as cubic B-splines. A cubic B-spline representation allowed to combine smoothing with the ability to evaluate derivatives, and hence vessel orientations, at any location, thus enabling the steps that follow [24] [25].

#### 3D reconstruction algorithm

The major stages of the 3D reconstruction algorithm can be listed as follows: (a) 3D centreline reconstruction; (b) 3D luminal cross-section reconstruction and (c) 3D luminal surface reconstruction.

3D reconstruction required definition of a 3D global reference system in which a 3D vessel will be generated. As the geometrical relationship between a radiographic plane (local reference system) and C-arm gantry can be easily described, we defined as global reference system the C-arm acquisition system (an exhaustive description of the C-arm system geometry can be found in [26]). The transformation from the local to the global reference system comprises a translation **t** followed by a rotation **R.**

Precisely, an initial estimate of the imaging geometry was obtained from the available image acquisition settings recorded by the X-ray system and stored in the file header of a radiographic image. Then, because small perturbation errors could affect the imaging geometry, i.e. possible table translation during the image acquisition or errors in the parameters, an optimization of the system geometry was necessary [27]. Corresponding anatomical landmarks, i.e. corresponding nodes extracted in previous step, were employed to refine the initial estimate [6]. With this approach, four corresponding landmarks were required (if no initial estimate is used and two projection images are at arbitrary and unknown orientation, determining the geometry requires eight or more corresponding landmark points [28], which can be unrealistic in many clinical cases). A 3D landmark point was reconstructed as the nearest point to corresponding non-intersecting projection lines [23]. Then, the obtained 3D locations were projected back onto a plane. The transformation, in the form of a rotation matrix **R** and a translation vector **t** that minimized the sum of distance errors of newly projected points from the input set of image coordinates was estimated using least-squares. Refinement of the geometry parameters was performed iteratively until convergence, such that at each iteration a new set of 3D landmark points was calculated using the newly computed transformation. For the cases in our study, we found that after 25 iterations, the accuracy improved relatively slowly with increasing number of iterations (after optimization, the maximum Root-Mean-Square (RMS) Euclidean distance between the set of image landmarks and corresponding projected reconstructed landmark points was 1.41mm, and the average RMS Euclidean distance was 0.85mm). Resulting transformation was applied to relative centrelines and profiles prior to 3D reconstruction.

The number of planes acquired in a routine ICA can occasionally vary from a minimum of two planes up to four planes, depending mostly on the lesion location and its visibility. It is important to make the method adaptable to a case in which more than two planes are acquired so to exploit all available information. If more than two projection images were available, 3D landmarks were reconstructed for all pairs of two projection images. Planes were sorted based on increasing RMS Euclidean distance between the set of image landmarks and corresponding projected 3D reconstructed landmarks, after optimization. According to the smallest RMS Euclidean distance, one plane was referred to as *reference* plane, and the pair of planes $P_{n,m}$ with the smallest RMS distance was referred to as *reference* pair and employed to generate a 3D centreline *C*. The imaging geometry of the *additional* planes was optimized with respect to the *reference* plane.

**Fig 3:**
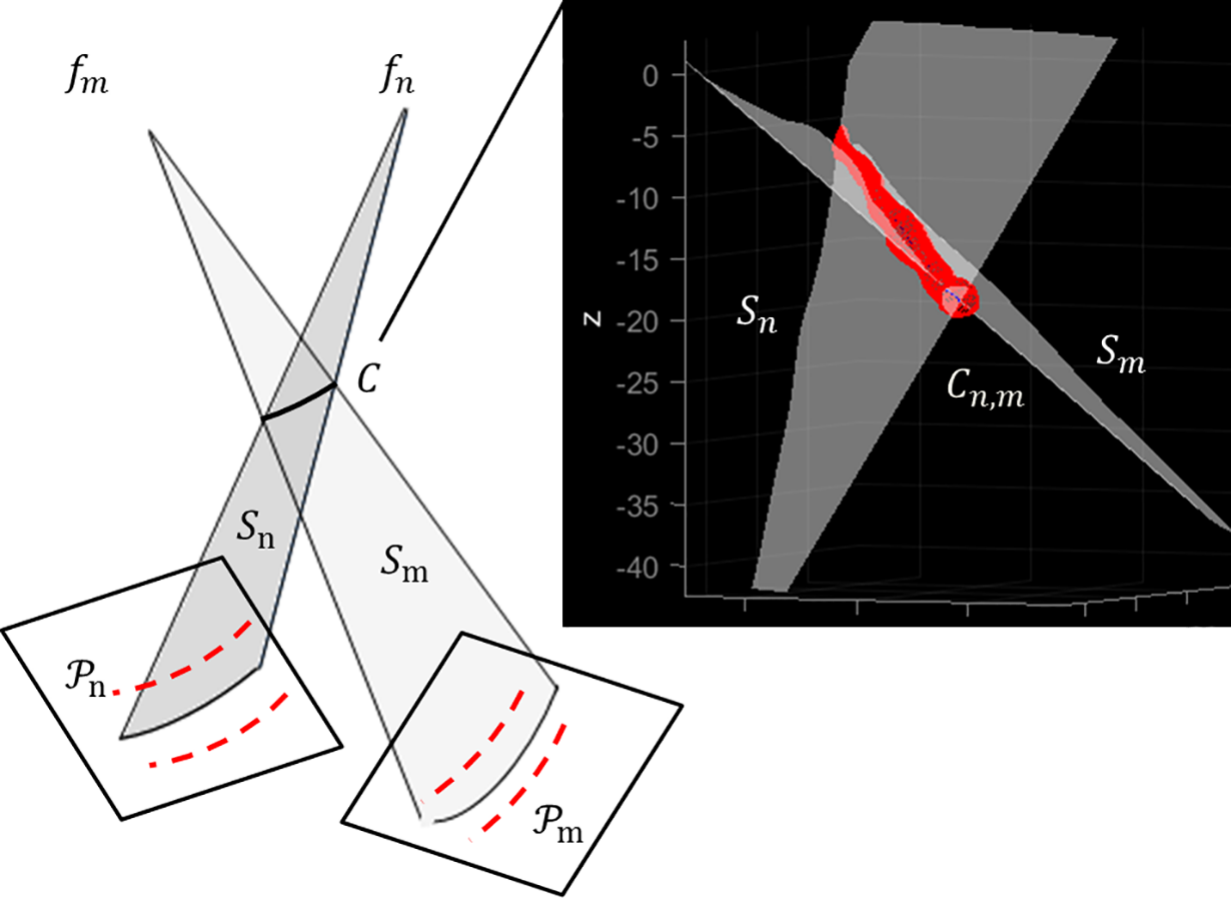
Intersection relationship of two corresponding surfaces for 3D reconstruction of an arterial segment centreline. The two triangulated surfaces *S*_*n*,*m*_ intersect at *C*.

**(a) 3D centreline reconstruction.** A 3D vessel centreline was reconstructed as intersection of surfaces defined by corresponding arterial segments. Notice that from step *1)* the segments correspondences between two projection planes $P_{n,m}$ were known. Given paired transformed centrelines *C*_*n*,*m*_ and their respective focal points *f*_*n*,*m*_, the surfaces *S*_*n*,*m*_ representing projectional beams for *C*_*n*,*m*_ were formed by connecting *C*_*n*,*m*_ to the respective focal point (Fig 3). The problem of finding the 3D centreline *C* from the projected curves *C*_*n*,*m*_ was thus reduced to finding the intersection curve of the two surfaces *S*_*n*,*m*_:

$$C=S_{n}\cap S_{m}$$ (1)

Precisely, *S*_*n*,*m*_ were triangulated surfaces and their intersection was calculated by applying the fast mutual triangle intersection test [29]. The basis of this test is that if two triangles (elements of the surfaces *S*_*n*,*m*_) intersect, then they overlap along the line of intersection of their respective planes. Briefly, the test computes the signed distance of the three vertices of a triangle from the plane containing the other triangle. If all the distances have the same sign, then the two triangles do not intersect. Otherwise, they may intersect, and the problem is reduced to an overlap test of two segments lying on the intersection line. The algorithm computes the scalar intervals for which the intersection line lies inside each triangle; if the intervals overlap, the intersection line segment is computed (i.e. two unique points). Robustness problems may rise when the triangles are nearly co-planar or an edge is nearly co-planar to the other triangle (especially when an edge is close to an edge of the other triangle). To handle these cases, a tolerance value was defined for the distances between the vertices of the two triangles by experimentally observing the highest 3D Euclidean distance between corresponding landmarks achieved i.e. if a point is relatively close to the other triangle’s plane, it is considered as being on the plane. All the intersections (line segments or single point) for the two triangulated surfaces S_n,m_ gave an ordered set of points that, connected, formed a 3D centreline *C*.

**Fig 4:**
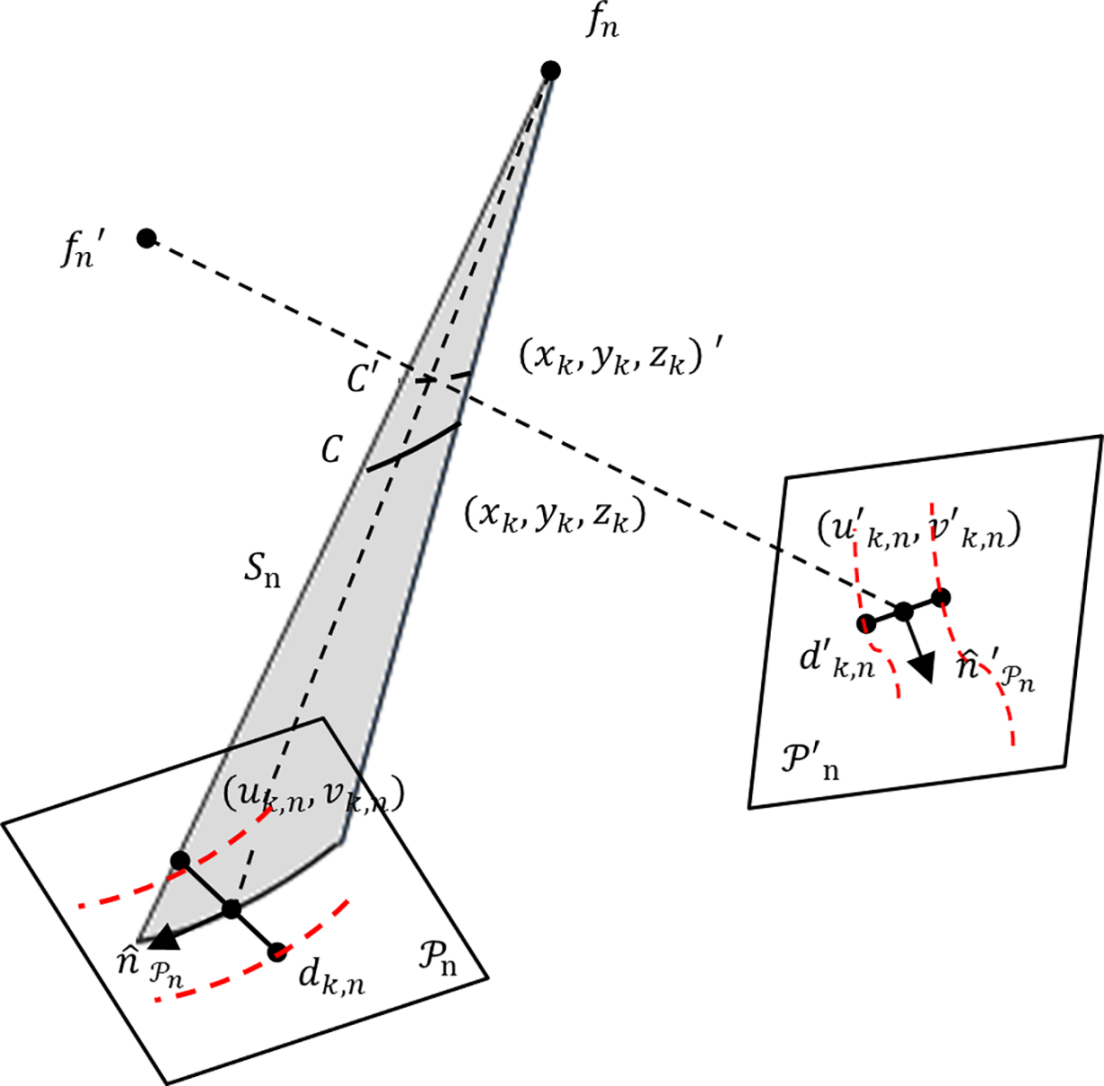
Intersection relationship of the *reference* plane $P_{n}$ with an *additional* plane ${P\prime }_{n}$. Diameter vector at (*x*_*k*_,*y*_*k*_,*z*_*k*_)′ is stored at (*x*_*k*_,*y*_*k*_,*z*_*k*_).

If the dataset included more than two projections, the pair of planes $P_{n,m}$ with the smallest RMS distance was employed to generate the *reference* 3D centreline *C*. To incorporate the information in vessel diameter from an *additional* image, the intersection of the *reference* surface with the *additional* surface was computed. A point (*x*_*k*_,*y*_*k*_,*z*_*k*_)′ on the newly computed intersection curve was associated to a point (*x*_*k*_,*y*_*k*_,*z*_*k*_) on the *reference* centreline *C* as the closest point to the projectional line *f*_*n*_, (*x*_*k*_,*y*_*k*_,*z*_*k*_) (Fig 4). Hence, the diameter vector information at (*x*_*k*_,*y*_*k*_,*z*_*k*_)′ was stored at the point (*x*_*k*_,*y*_*k*_,*z*_*k*_) for next 3D luminal cross-section reconstruction.

**(b) 3D luminal cross-section reconstruction.** In 3D reconstruction from X-ray angiograms, the accuracy of the luminal contour will depend on the number *N* of available projection planes, their mutual orientation and their orientation with respect to the arterial segment of interest. Due to the limited number *N* of projection planes, a conic function could provide an acceptable approximation for luminal contours. A visual inspection of various OCT datasets indicated that healthy arterial segments have nearly circular luminal cross-sections. However, when a build-up of plaque occurred, the circular contour deformed in various and unpredictable ways: in some cases, the plaque distribution was uniform along a cross-section, and the lumen still appeared as roughly circular, in other cases the distribution was less uniform, yielding various shapes (e.g. semi-circular, oval, triangular). Samples of OCT cross-sections from diseased arterial segments are reported in the next section. Based on such observations, design of a luminal cross-section consisted of two stages: first, a cross-section was designed using the circularity approximation to handle the limited number of projections, and then, local adjustment of the initial cross-section was carried out in accordance with 2D projected information (2D boundaries), as follows.

**Fig 5:**
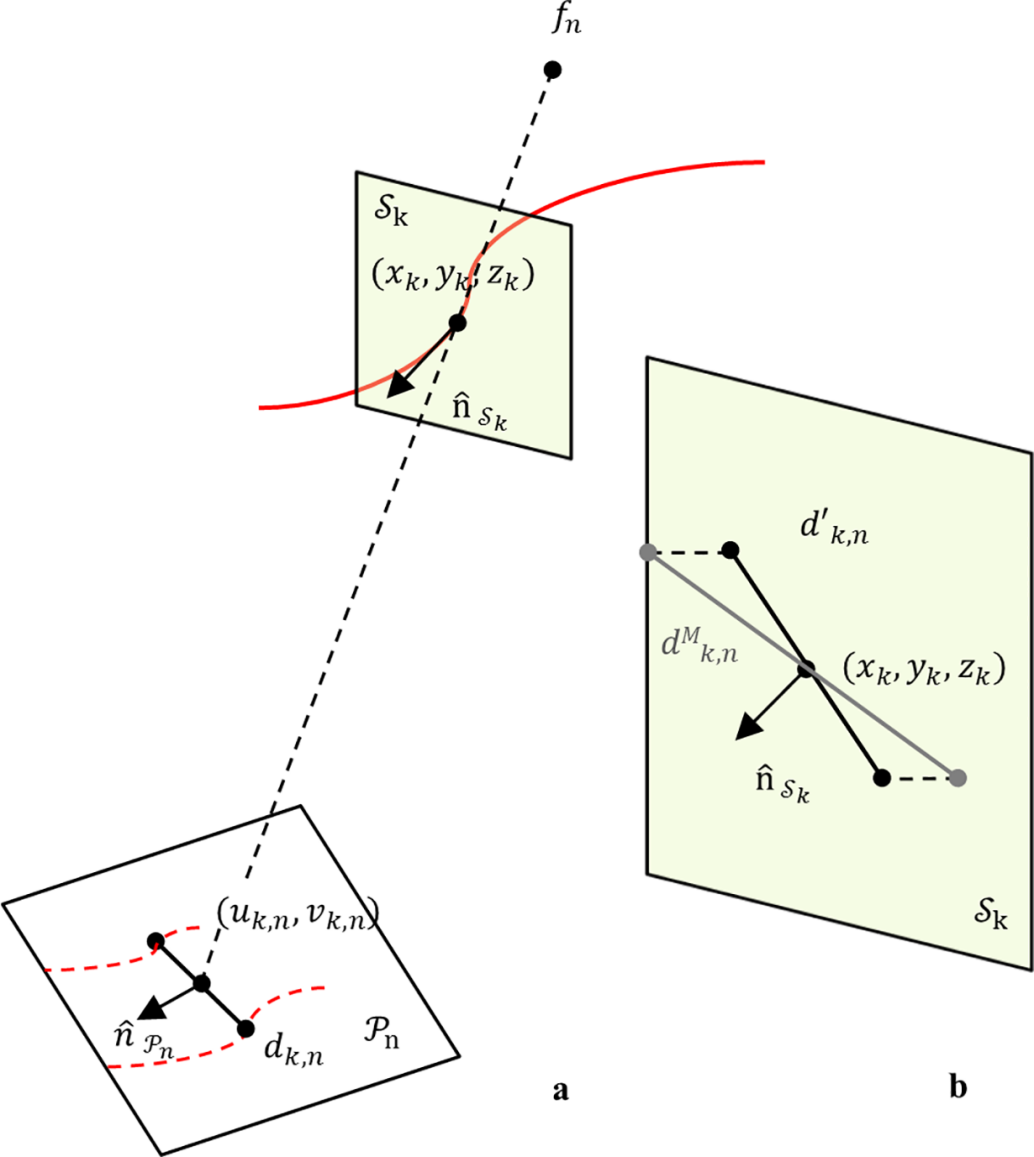
3D luminal cross-section reconstruction. a) Schematic view of a projection plane $P_{n}$, correspondence of a 3D centreline point; definition of cross-sectional plane $S_{k}$. b) 3D reconstruction of a diameter vector *d*′_*k*,*n*_; magnification, followed by projection onto the cross-sectional plane $S_{k}$.

The computed 3D centreline *C* was the spine along which the lumen cross-sections were generated. Cross-sectional planes were defined in 3D space at equidistant points *k* along a 3D reconstructed centreline curve *C*. Given a 3D point (*x*_*k*_,*y*_*k*_,*z*_*k*_) on a centreline curve *C*, a cross-sectional plane $S_{k}$ was described by the tangential vector ${\hat{n}}_{S_{k}}$ of *C* at (*x*_*k*_,*y*_*k*_,*z*_*k*_) (Fig 5A). 2D image point (*u*_*k*,*n*_, *v*_*k*,*n*_) was the projection point of (*x*_*k*_,*y*_*k*_,*z*_*k*_) on a projection plane $P_{n}$, and *d*_*k*,*n*_ was the diameter vector orthogonal to the 2D vessel centreline direction at (*u*_*k*,*n*_, *v*_*k*,*n*_). The vector *d*_*k*,*n*_ was then scaled by the magnification factor, computed as the ratio of the distances *d*(*f*_*n*_, (*x*_*k*_,*y*_*k*_,*z*_*k*_))/*d*(*f*_*n*_, (*u*_*k*,*n*_, *v*_*k*,*n*_)), with *f*_*n*_ the focal point. Generally, the diameter vector *d*_*k*,*n*_ was not orthogonal to the 3D centreline curve *C* at (*x*_*k*_,*y*_*k*_,*z*_*k*_); thus, the scaled diameter $d_{k,n}^{M}$ was projected on the cross-sectional plane $S_{k}$ at (*x*_*k*_,*y*_*k*_,*z*_*k*_) as (Fig 5B) [23]:

$${{d\prime }_{k,n}=d}_{k,n}^{M}\text{-}\;(d_{k,n}^{M}∙{\hat{n}}_{S_{k}}){\hat{n}}_{S_{k}}.$$ (2)

At this stage, a luminal contour was designed as a circle, with diameter given by the average length of reconstructed diameter vectors $d_{k}^{\prime }$.

We used Non-uniform Rational Basis Splines (NURBS) to parametrize a luminal contour. Parameterization using NURBS allowed us to obtain a flexible and versatile modelling of a cross-section, capable of representing simple curves (e.g. circles) and more complex free-form ones. A luminal contour was represented as:

$$\gamma_{k}(u)=\sum_{i=0}^{n}B_{i,p}(u)Q_{i}\;u\in [0,1],$$ (3)

where *Q*_*i*_ were the control points, *n+1* was the number of control points, *p* was the degree of the curve, and *B*_*i*,*p*_(*u*) was a rational basis function of *i*^*th*^ control points and *p*^*th*^-degree, defined on the knot vector. For a comprehensive treatment of NURBS, refer to Piegl and Tiller [16].

**Fig 6:**
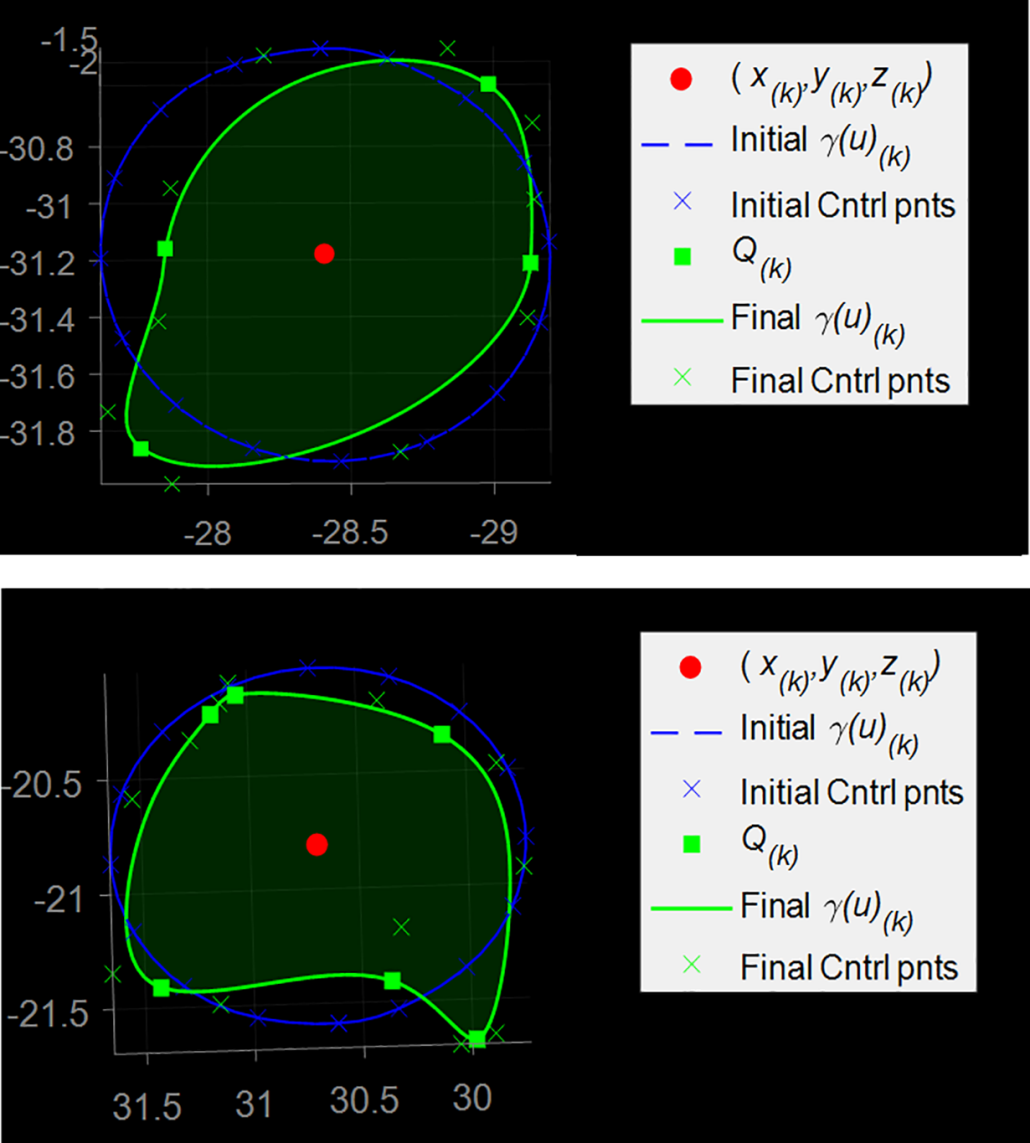
3D luminal contour reconstruction. An initial circular luminal contour on a cross-sectional plane (diameter *d*′_*k*,*N*_) and its control points; four (top) and six (bottom) interpolatory control points Q_k_ are reconstructed; the initial control points are adjusted as described in the text; the final contour is obtained.

Initially, a circular luminal contour *γ*_*k*_(*u*) was uniformly sampled along its length; the order of a curve *γ*_*k*_(*u*) was three and n+1 control points were uniformly distributed along its length (*n+1* = 17 control points were found to be suitable for all our cases with *2N = 4*,*…*, *8* reconstructed 3D boundary points). The shape of the curve was then adjusted as follows. From Eq 2, a set of two 3D coordinate points describing a 3D luminal cross-section at (*x*_*k*_,*y*_*k*_,*z*_*k*_) was obtained per plane. Given *N* projection planes, *Q*_*k*_ = 2*N* points describing a 3D luminal cross-section at (*x*_*k*_,*y*_*k*_,*z*_*k*_) were obtained. 3D points *Q*_*k*_ were labelled as interpolatory control points for the luminal contour *γ*_*k*_(*u*). For each *Q*_*k*_, the Euclidean distances from the control points of *γ*_*k*_(*u*) were computed. Each point *Q*_*k*_ replaced its closest control point. To achieve interpolation at a point *Q*_*k*_, the multiplicity of its knot value was increased (more precisely, a knot value was repeatedly inserted until its multiplicity was equal to the degree of the curve) [16]. The remaining control points of *γ*_*k*_(*u*) were labelled as non-interpolatory. In order to avoid unrealistic spikes, a neighbouring control point of an interpolatory point was removed if the difference between their radial distances was at least 25% of the contour mean radii. Otherwise, *γ*_*k*_(*u*) was locally adjusted by spacing apart the knots of non-interpolatory control points. Fig 6 shows two examples of initial contour (blue curve), computed interpolatory points Q_k_ (green squares), and resulting adjusted contour γ_k_(u) (green curve).

**Fig 7:**
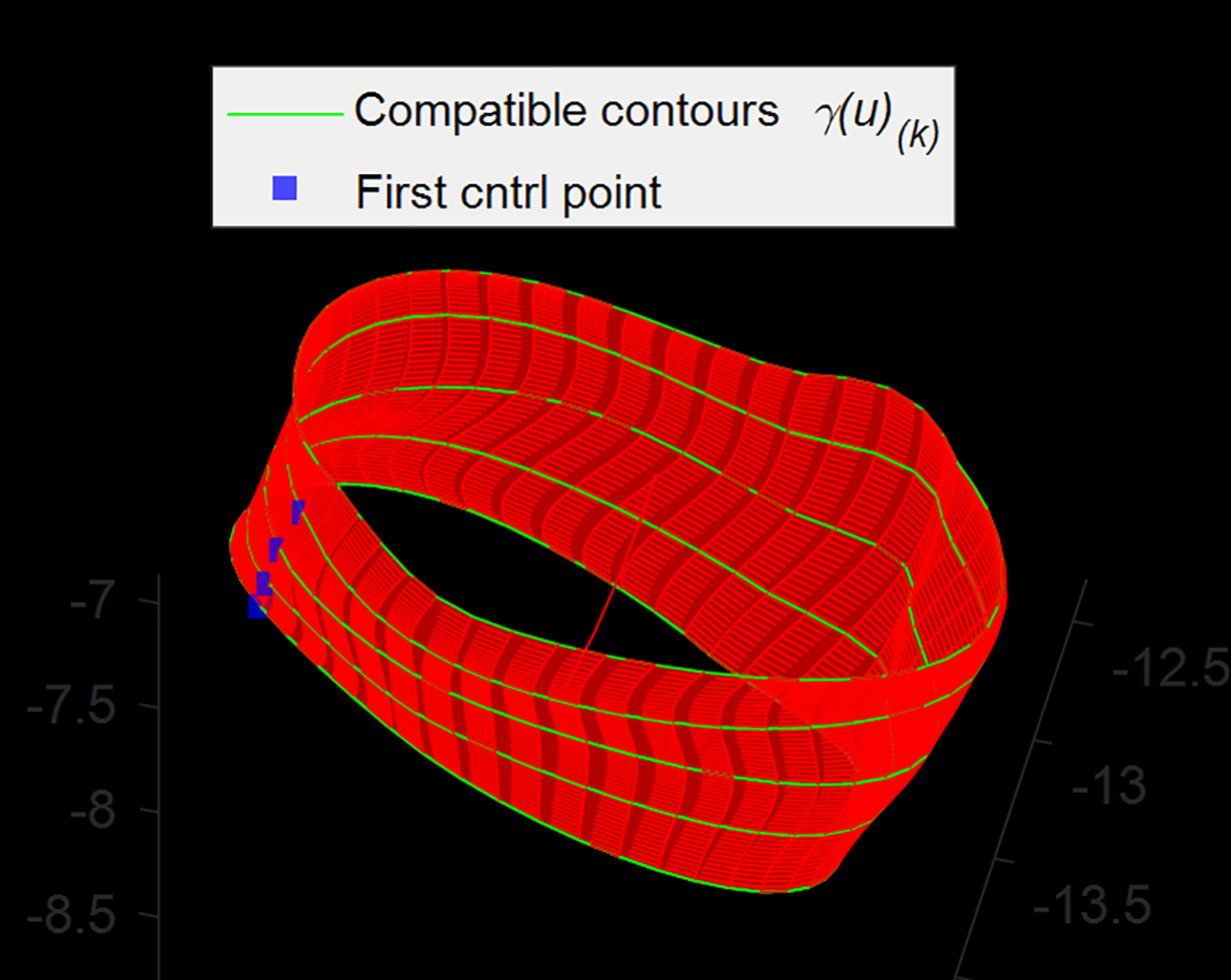
3D luminal surface reconstruction. A subset of contours (green) is shown; red surface is the lofted surface. Alignment of the first control points (blue) was required to avoid torsions.

**(c) 3D Surface reconstruction.** A technique called *lofting* (or *skinning*) can be used to generate the surface passing through a set of luminal contours {*γ*_*k*_(*u*)}, *k* = 0,…,*K* [16]. Lofting was performed as an interpolation across the control points in each luminal contour, yielding the new control points *P*_*j*_ of the lofted vessel surface (Fig 7).

In order to preserve continuity in the luminal surface, some precautions were taken prior to interpolation across contours. Control points were sorted according to their angles on the plane and in the same direction (clockwise), so that the luminal surface had no flips or twists. In addition, the set of first control points of all luminal contours formed a continuous curve; without this precaution, regions of torsion in the luminal surface would appear in correspondence of discontinuities along the curve.

Importantly, lofting required that the curves were compatible, that is they had common degree and number of control points and were defined over the same knot vector [30]. To ensure compatibility, degrees were elevated to a maximum degree, then knot vectors were merged, and finally knots were inserted so that contours had the same knot vector and, hence, the same number of control points [31]. This process did not change the shape of luminal contours.

Then, the parameters $\left\{{\bar{v}}_{k}\right\}$ and the knot vector *V* were computed [16], and they were used to interpolate degree-*q* curves through the control points of Eq 4:

$$\gamma_{i}(v)=\sum_{j=0}^{m}B_{j,q}(v)P_{j},$$ (4)

so that Eq 5 interpolated *Q*_*i*_ at certain *v* values. These newly obtained control points were the control points of the lofted surface (luminal vessel surface)

$$L(u,v)=\sum_{i}^{n}\sum_{j}^{m}B_{i,p}(u)B_{j,q}(v)P_{i,j},$$ (5)

defined over the knot vectors *U* and *V*. By applying the lofting technique, 3D luminal vessel surfaces were reconstructed as in Eq 5.

### Dataset

#### Study population

The study population comprised patients enrolled in clinical studies at the catheterization laboratory, of the John Radcliffe University Hospital, Oxford, UK. Ethics approval (Oxford Regional Ethics Committee) was obtained and all patients provided informed written consent for all study procedures. OCT cohort was enrolled from the *Plaque Imaging and Biomarker Study—*Ethics Reference 08/H0603/41. Patients who were scheduled for non-emergency coronary angiography with the view to possible percutaneous coronary intervention were recruited. The angiographic images were acquired prior to inserting the intracoronary imaging catheter. OCT of the diseased coronary artery segment was performed prior to the deployment of any stent. The FFR cohort was part of the *Oxford Acute Myocardial Infarction Study (OxAMI)*—Ethics Reference 10/H0408/24. Patients were recruited in 2010/2011 for the Biomarkers Study (OCT cohort) while the OxAMI study (FFR cohort) is still ongoing (patients were selected in 2013/2014.) In this study, results are obtained on twelve patients. Six patients underwent ICA and OCT; six patients underwent ICA and FFR.

#### Data acquisition

X-ray arteriographic images were recorded with single-flat-panel mobile C-arm (Axiom Artis, Siemens; contrast agent Niopam, Bracco), at 15 frames/sec, with the detector at angles selected by the interventional cardiologist. Each frame represents a projection of the coronary tree onto the single-plane system. The frames were related to the cardiac cycle by electrocardiographic (ECG) gating, which enabled selection of views at the same cardiac phase. Image acquisition settings, including focal point to image plane distance, field of view, image plane orientation, image pixel spacing, were automatically stored with each image file in DICOM format.

Serial OCT images were acquired using the Ilumien system (St. Jude Medical, Minneapolis, MN, USA). Optical Coherence Tomography uses back-reflected infrared light to perform *in-situ* micron scale tomographic imaging of the vessel anatomy and internal microstructure of plaques [32,33]. With manual injection of 20ml of contrast solution, the OCT catheter (2.7 F) was automatically pulled back at speed of 20mm/sec and rate of 100 frames per second spanning approximately 50mm (a high frame-rate is desirable for many reasons, including higher longitudinal resolution and cardiac motion-free intracoronary imaging [34,35]).

Fractional Flow Reserve is defined as the ratio between the distal pressure *P*_*d*_ (measured close to the distal stenosis) and the proximal pressure *P*_*a*_ (equivalent to mean aortic pressure) of the stenosis [36]. A cut-off value of 0.8 indicates ischemia. Calibrated and equalised pressure wire (Certus, St Jude Medical, St Paul, MN, USA) was advanced to cross the lesion. Hyperaemia was induced using an intravenous infusion of adenosine at a rate of 140 μg/kg/min. Measurements of absolute mean aortic pressure, *P*_*a*_, and mean distal pressure, *P*_*d*_, were recorded when reaching steady-state hyperaemia (under the assumption that during hyperaemic condition, myocardial resistances are minimal and remain constant).

### Experiments

In order to investigate the accuracy of the proposed method, a series of experiments were performed on routine clinical data.

#### Assessment of 2D vessel detection

Accurate vessel extraction is crucial to accurate 3D vessel reconstruction. Obtained vessel boundaries were compared with expert manual tracing, in terms of reduction in diameter, *R*, due to a lesion (stenosis severity):

$$R=\frac{\bar{D_{d},D_{p}}-D_{o}}{\bar{D_{d},D_{p}}},$$ (6)

where *D*_*o*_ is the minimum occluded diameter, *D*_*p*_ and *D*_*d*_ are the proximal and distal reference diameters, respectively ($\bar{D_{d},D_{p}}$ is their average value). 2D expert manual tracing was performed off-line on each radiographic image using apposite 2D-QCA software (McKesson Cardiology). The profiles of a segment of interest were traced to measure the above quantities of interest (calibration was performed automatically by means of the size of the size of the guiding catheter).

#### Assessment of 3D reconstruction

OCT images were analysed off-line by one author (MA) who was completely blinded to the 3D reconstruction method. Analysis was performed using specialist software Ilumien Analysis Package (St. Jude Medical). Manual measurements of lumen mean, minimum, and maximum diameters and cross-sectional areas were done on every individual frame of the lesion (calibration was performed automatically by means of the size of the OCT catheter).

The comparison of the OCT analysis and corresponding 3D reconstructed segment analysis demanded matching of the two modalities. First, corresponding segments were visually identified on OCT frames and 2D angiograms. Then, a common anatomical landmark was identified on both imaging modalities (e.g. a side branch or the ostium for the main epicardial vessels); from the landmark, a segment length of interest was established based on OCT data. Cross-sectional areas were computed along this length, for both OCT and 3D reconstructed data (with sampling rate as OCT).

Comparison with OCT derived analysis involved the proposed method and conventional approaches, namely *i)* circular cross-section approximation (with diameter *d*_*k*,*n*_) and *ii)* ellipse fitting of the computed 3D profile points *Q*_*k*_. A least-squares method was employed that minimized the Euclidean distances of the computed *Q*_*k*_ = 2*N* points to the ellipse [37]. Comparison was carried out by computing, for each approach, the difference in every pair of corresponding cross-sections between OCT and the approach, followed by a RMS of all the discrepancies derived from every individual pair of cross-sections.

For a 3D reconstructed segment, luminal cross-sectional areas were calculated using Stokes Theorem for computing the area of a planar polygon [38]. Parametric points {*p*_*i*_ = (*u*_*i*_,*v*_*i*_)}, *i* = 0, …, *n* on a luminal cross-sectional curve were computed following a uniform distribution, and the area *A* enclosed by the curve was calculated as:

$$A=\frac{1}{2}\sum_{i=0}^{n}\left(u_{i}v_{\left(i+1\right)}-u_{\left(i+1\right)}v_{i}\right).$$ (7)

**Comparison with FFR measurements.** Progressively increasing plaque burden causes increasing severity of luminal stenosis and can lead to hemodynamically restriction of flow across a lesion [39]. ICA-derived luminal measurements might be employed for the prediction of hemodynamically significant stenosis as determined by FFR [40,41]. Correlation between the FFR measurement (i.e. derived from pressure gradient across a stenotic lesion) and the volume reduction of that lesion, as calculated with the proposed 3D reconstruction method, was investigated. [18].

For comparison, the matching segment was visually identified on radiographic images, acquired while performing the FFR procedure, as between the proximal location of the pressure sensor and its distal location. The same segment was identified on corresponding diagnostic 2D angiograms.

The reduction in volume was computed as the ratio of the real volume (*V*_*R*_) to the interpolated volume (*V*_*I*_), as if there were no lesion between the proximal and the distal portions of a segment of interest. As before, a set of luminal cross-sectional areas along a segment was computed by applying Eq 7. For the interpolated lumen, cross-sections were assumed circular and diameters were estimated through linear interpolation between proximal and distal diameters.

## Results

The algorithm was implemented in MATLAB (The MathWorks Inc., Natick, MA, USA). 3D reconstruction and analysis were performed off-line after the completion of angiographic study, using an Intel Core 2 i7-480MQ CPU @ 2.70 GHz, with a computational time of 1–2 min. In this section, coronary artery segments for a patient are abbreviated as: LCx, Left Circumflex; LAD, Left Anterior descending; Marg, Marginal; IntM, Intermediate; RCA, Right Coronary Artery.

**Fig 8:**
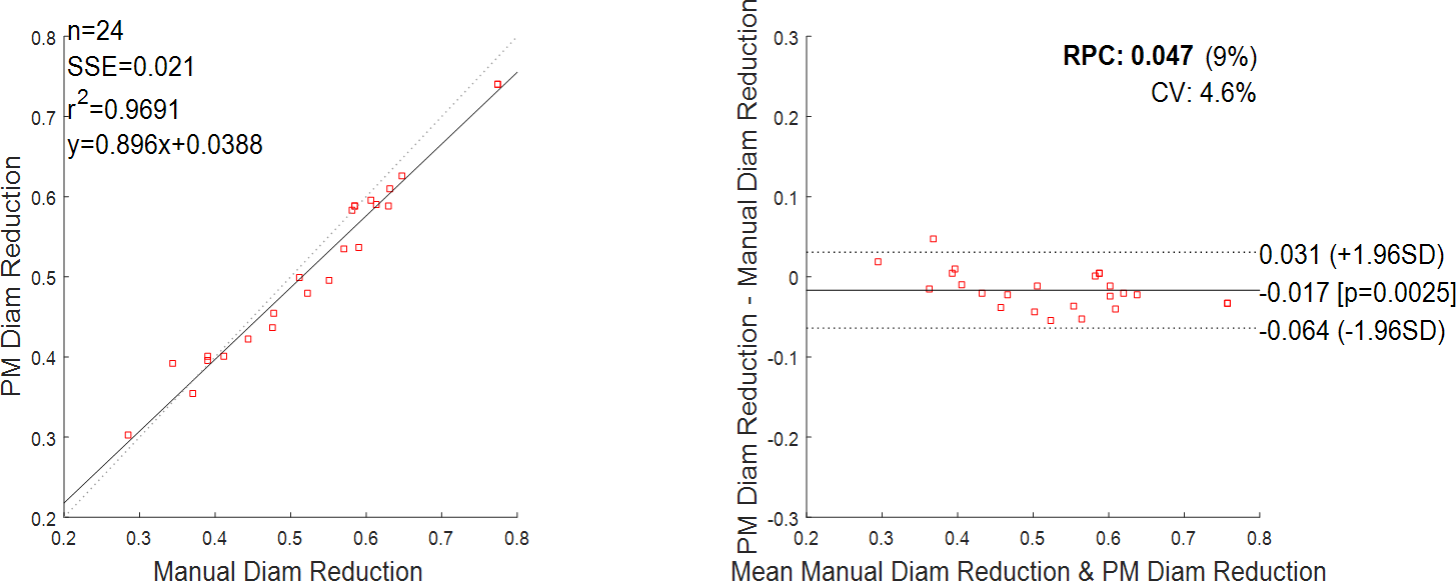
Reduction in diameter, comparison between manual and semi-automatic detection (n = 24 data). On the left, the regression line between the two methods. Regression line has a slope of 0.869 and an intercept of 0.0388. Pearson r-value squared is r^2^ = 0.9691. Sum of the squared errors is SSE = 0.021. On the right, the Bland-Altman plot. Reproducibility coefficient and % of mean values RPC (%) = 0.047 (9%); coefficient of variation CV = 4.6%. The solid line represents the mean of the differences; dotted lines define the interval mean difference ± 1.96 SD.

The results of the 2D detection are given in Fig 8. A total of 24 stenotic segments from 12 patients were included to compare reduction in diameter by the fully manual and the semi-automatic segmentation method. The diseased segments had various degrees of severity: the average reduction in diameter was of 53.2% as assessed by the manual segmentation, the minimum reduction was 28.5%, and the maximum reduction was 77.4%. There was an excellent correlation between the manual segmentation and the implemented method, with a coefficient of determination r^2^ = 0.969 (Fig 8, left). The Bland-Altman plot (Fig 8, right) also depicts a high agreement between the manual and semi-automatic driven values with a mean difference close to zero and with only one value outside the 1.96 * SD cut-offs (SD = standard deviation, value corresponding to 55.0% reduction as assessed by manual segmentation against 49.6% as assessed by the implemented method).

**Fig 9:**
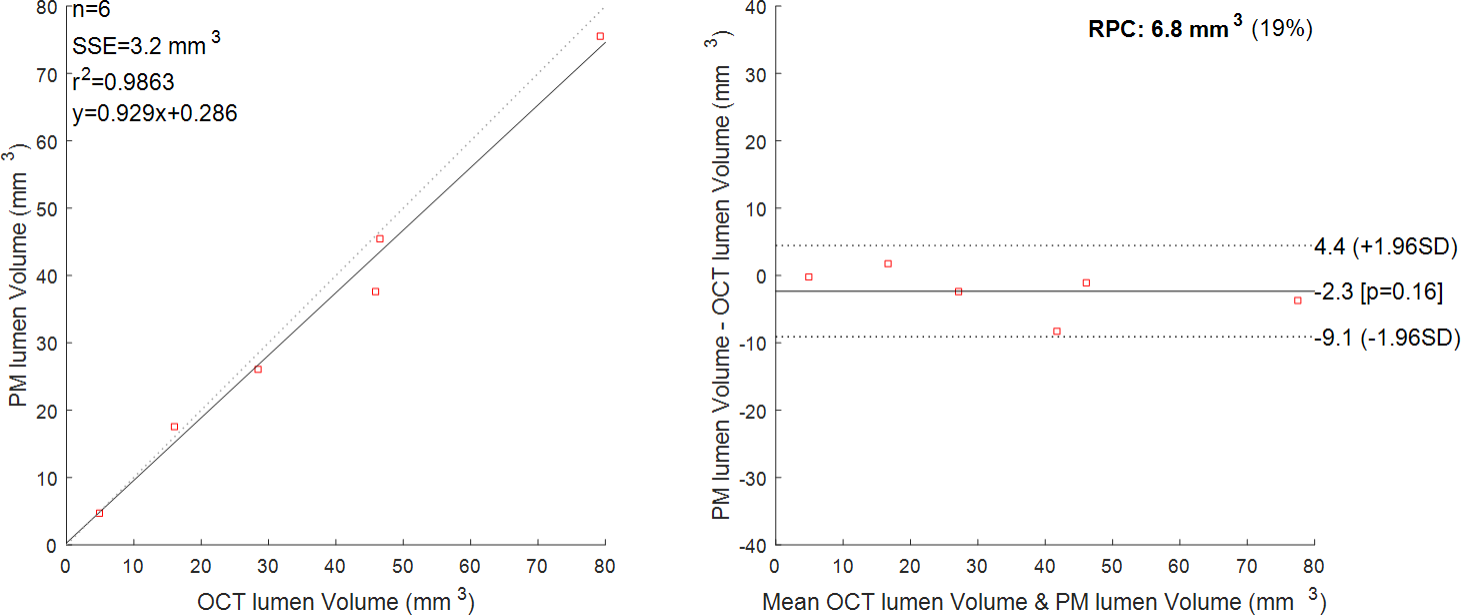
Luminal volume, comparison between the proposed method (PM) and OCT. n = 6 patients. On the left, the regression line between the two methods. Regression line has a slope of 0.929 and an intercept of 0.286. Pearson r-value squared is r^2^ = 0.9863. Sum of the squared errors is SSE = 3.2 mm^3^. On the right, the Bland-Altman plot. Reproducibility coefficient and % of mean values RPC (%) = 6.8(19%). The solid line represents the mean of the differences; dotted lines define the interval mean difference ± 1.96 SD.

A total of 6 diseased segments from 6 OCT patients (one segment per patient) were examined (LAD = 2, RCA = 1, Marg = 1, IntM = 1, LCx = 1). The number of analysed cross-sections varied between 18 and 134 per patient, this depending on the acquired OCT data (i.e. quality of the OCT run, lack of anatomical landmarks, lesion length). The computed volume of the lumen based on the OCT analysis was compared to the computed volume of the lumen based on the proposed 3D reconstruction method, for corresponding segments. The results show an excellent correlation between the two methods with a coefficient of determination r^2^ = 0.986 (Fig 9, left). Bland-Altman plot (Fig 9, right) confirmed the high agreement between the two methods with all the values being within the 1.96 * SD cut-offs. Corresponding quantitative data are presented in Table 1. Mean diameter, minimum diameter, maximum diameter are also reported, with a maximum distance error of 0.79mm and a mean error of 0.29mm. Overall, Patient 1 (LAD) shows the highest percentage error in volume (18.10%) and in maximum diameter (24.18%), while Patient 3 (Marg) shows the highest percentage error in minimum diameter (28.59%). For these patients, we observed a lower contrast in correspondence of the lesion segment (possible thrombus), which can result in slight underestimation of the diameter.

**Table 1:** Results of the analysis with OCT and proposed method (PM).

Patient	Method	Epicardial artery	Volume (mm^3^)	Estimated Stenosis (%)	Mean diam (mm)	Min diam (mm)	Max diam (mm)
	OCT	LAD	45.89	23.03	2.09	1.00	3.28
1	PM		37.58	27.91	1.87	1.11	2.49
	Err (%)		18.10		10.55	11.36	24.18
	OCT	RCA	46.51	66.32	1.91	1.03	2.65
2	PM		45.50	66.64	1.94	0.84	2.75
	Err (%)		2.16		1.51	18.23	3.78
	OCT	Marg	28.39	28.91	1.65	0.80	2.68
3	PM		26.08	21.25	1.61	0.57	2.65
	Err (%)		8.15		2.45	28.59	1.07
	OCT	IntM	79.27	51.05	1.78	0.77	2.91
4	PM		75.58	48.01	1.98	0.82	3.59
	Err (%)		4.66		11.15	7.10	23.33
	OCT	LCx	4.97	44.08	1.33	0.81	1.90
5	PM		4.66	47.80	1.36	0.86	1.92
	Err (%)		6.26		2.18	5.56	1.16
	OCT	LAD	15.92	76.82	1.74	0.79	2.97
6	PM		17.62	72.70	2.05	0.65	3.07
	Err (%)		10.64		17.68	18.18	3.51

**Fig 10:**
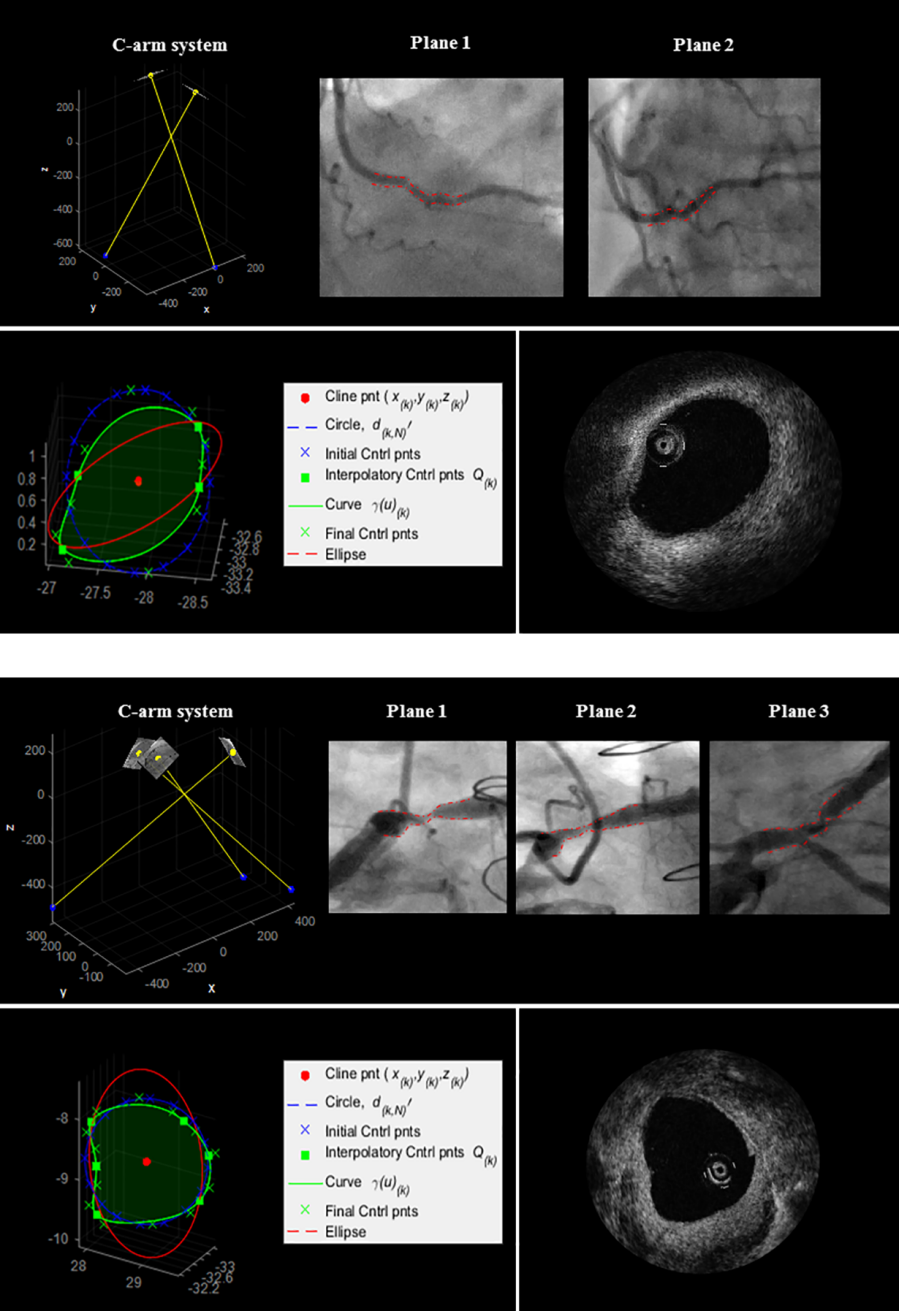
3D reconstruction of Patient 1 and Patient 2 (Table 2). Top: 2D angiographic images and their 3D relationship. Bottom: an example of 3D reconstructed luminal cross-section (green), circular fitting (blue), and elliptical fitting (red); corresponding OCT frame.

Analysis of paired cross-sectional areas involved the proposed method, circular approximation and ellipse fitting. Two cases of diseased luminal cross-sections, acquired with OCT and as reconstructed with the three approaches, are shown in Fig 10. The reconstructed luminal cross-sections were found to be a closer representation of the true OCT contours both visually and quantitatively. Maximum and minimum discrepancies, standard deviation and RMS discrepancy of cross-sectional areas are reported in Table 2. The proposed method showed the lowest RMS discrepancy, with overall values ranging from 0.213mm^2^ to 1.013mm^2^, and maximum error of 1.837mm^2^. Maximum errors with circular and elliptical fitting were, respectively, 2.322mm^2^ and 5.078mm^2^.

**Table 2:** Comparison of proposed method (PM), circular and elliptical fit.

Patient	Method	Number of Cross-sections	Max Err	Min Err	St Dev	RSME
			(mm^2^)	(mm^2^)	(mm^2^)	(mm^2^)
	PM		1.837	0.077	0.508	1.013
1	Circle	101	2.322	0.079	0.697	1.280
	Ellipse		5.078	0.112	1.098	1.788
	PM		1.074	0.066	0.287	0.669
2	Circle	81	1.223	0.015	0.387	0.734
	Ellipse		2.734	0.385	0.606	1.042
	PM		0.667	0.013	0.193	0.395
3	Circle	58	0.716	0.122	0.164	0.560
	Ellipse		3.676	0.001	0.942	1.223
	PM		1.860	0.033	0.466	0.875
4	Circle	134	1.941	0.050	0.507	0.944
	Ellipse		1.952	0.105	0.508	0.909
	PM		0.327	0.028	0.096	0.213
5	Circle	18	0.764	0.011	0.194	0.332
	Ellipse		1.415	0.117	0.371	0.872
	PM		0.735	0.150	0.187	0.429
6	Circle	29	1.075	0.091	0.231	0.822
* *	Ellipse		2.171	0.043	0.479	0.822

**Fig 11:**
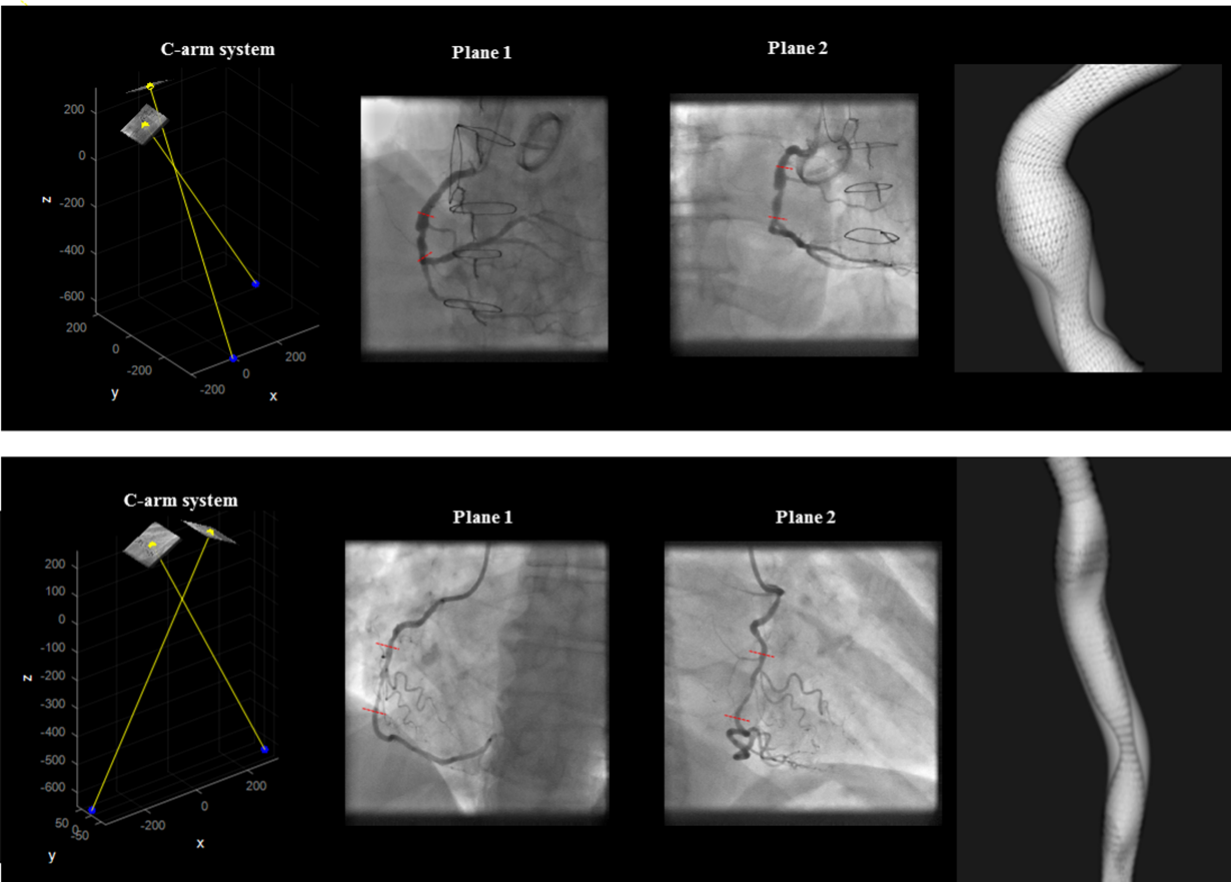
3D models of a lesion. 2D angiographic images and their 3D relationship; 3D reconstructed model and interpolated model (shaded grey region). The illustrated cases reported an FFR value equal to 0.75 and 0.89, respectively.

**Fig 12:**
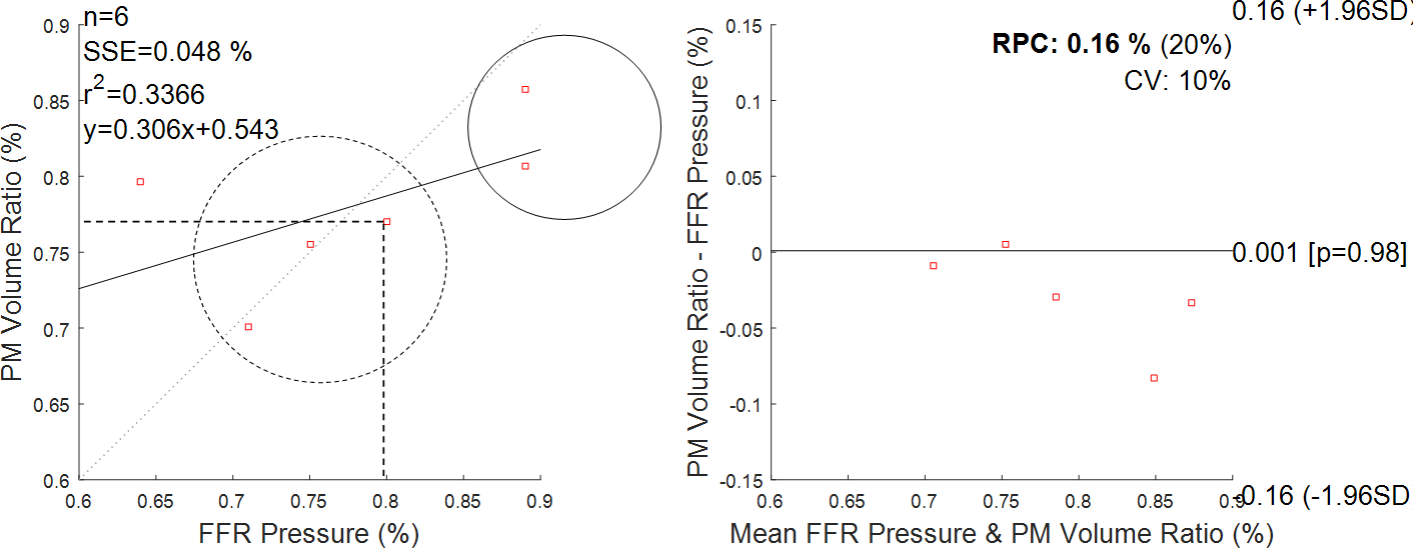
Volume reduction as from proposed method (PM) and measured pressure gradient (FFR measurement). On the left, the regression line between the two methods. Dashed circle encloses positive FFR clinical outcomes; solid circle encloses negative FFR clinical outcomes. Dashed lines indicate that the cut-off of 0.8 in FFR corresponds to a cut-off of 0.77 in volume drop. On the right, the Bland-Altman plot. Reproducibility coefficient and % of mean values RPC (%) = 0.16% (20%); coefficient of variation CV = 10%. The solid line represents the mean of the differences.

A total of 6 diseased segments from 6 FFR patients were analysed (LAD = 3, RCA = 2, Marg = 0, IntM = 0, LCx = 1). Fig 11 illustrates two 3D reconstructed lesions, their interpolated volume and proximal and distal pressure measurement locations. The measured FFR values were compared to segment volume reduction, *V*_*R*_*/V*_*I*_, calculated based on the 3D reconstructed lesion. The results are illustrated in Fig 12. An FFR measurement of 1.0 indicates normal blood flow, an FFR measurement lower than 0.8 is the clinically validated cut-off that is used in clinical practice to indicate a functionally significant lesion that may cause ischemia. FFR measurements varied between 0.64 and 0.89, and 3/6 lesions were evaluated as physiologically significant. The results showed a good correlation between FFR and estimated volume reduction, *V*_*R*_*/V*_*I*_, for 5/6 patients. However, volume reduction for Patient 6 seemed to indicate a not severely obstructive stenosis, while the FFR measurement equal to 0.64 indicates a clinically significant stenosis.

## Discussion and conclusions

This paper proposes a novel method to generate in an effective manner a 3D computational patient-specific model of lesioned vessel, directly from 2D projections acquired while performing invasive coronary X-ray angiography, and appropriate for immediate geometric and quantitative analysis. The technical novelty of our work resides in the 3D reconstruction strategy and the cross-section NURBS parameterization. The proposed method i) computes a 3D centreline as intersection of surfaces defined by corresponding branches, overcoming the issues related to point-to-point matching based reconstruction; ii) it parametrizes a 3D luminal cross-sectional contour by using NURBS, and adjusts a model to interpolate computed contour points of the luminal surface, thus avoiding the tubular approximation; iii) it generates a 3D model by lofting of the obtained cross-sections, thus it allows us to generate any complex shape from any set of cross-sections. Importantly, the method is not restricted to a specific number of 2D projections and additional information can be integrated sequentially.

The design of a 3D cross-section consisted of two main steps: first, a template contour was built on a cross-sectional plane with the circularity approximation and, second, an alteration of the contour was performed accordingly to the computed 3D luminal contour points. In the first step, the use of 3D cross-sectional planes circumvents the problems with degenerated ellipses and non-parallel neighbouring cross-sections [14]. Importantly, the use of a template contour handles the limited number of projections in ICA, often as low as two, i.e. lack of information on the vessel boundary. The choice of a circular template contour derived from the observation on OCT data that healthy cross-sections are roughly circular. When computing the diameter vectors, i.e. 3D lumen boundary points, we accounted for the fact that the projections are in general not perpendicular to the normal of the cross sectional planes of the centreline [5,8]. In the second step, parameterization of the luminal contour by means of NURBS functions permitted a smooth representation of the contour, and control over changes on its form. Initial control points of the template circular contour were adjusted or deleted so that the contour curve interpolated the computed points representing 3D lumen boundary points. The influence on the shape of the initial control points depends on their location with respect to the interpolatory control points. Generally, for a higher number of acquired projections, i.e. more information on the luminal surface is provided, their influence will decrease, while for two projections only, their influence will be higher.

Validation of the 3D reconstruction method involved comparison with independent clinical measurements that are used routinely in the clinical assessment of coronary artery stenosis severity. To the best of our knowledge, no previously proposed method was validated using OCT data. Importantly, clinical data included stenoses of various degree of severity and comparison was performed on a frame-to-frame basis. This comparison is of great clinical value, as it suggests that 3D renderings of coronary arteries from ICA could help assessment of coronary artery stenosis severity, in combination *or* without the need for expensive and potentially hazardous invasive techniques.

The measured lumen volumes based on the OCT analysis were similar to the computed lumen volumes based on the 3D reconstruction method, indicating a very good performance of the algorithm. For the six patients, the maximum difference in minimum diameter and maximum diameter values were about 29% and 24%, respectively. For these two cases, we observed a lower image contrast in correspondence of the lesion segment, possibly due to a thrombus, which could result in an underestimation of the diameter. Correlation of volumes showed very good correspondence between our proposed method and OCT; however, the relative errors in diameter between our method and OCT are likely to be underestimated as they are averaged along the length of the segment.

**Fig 13:**
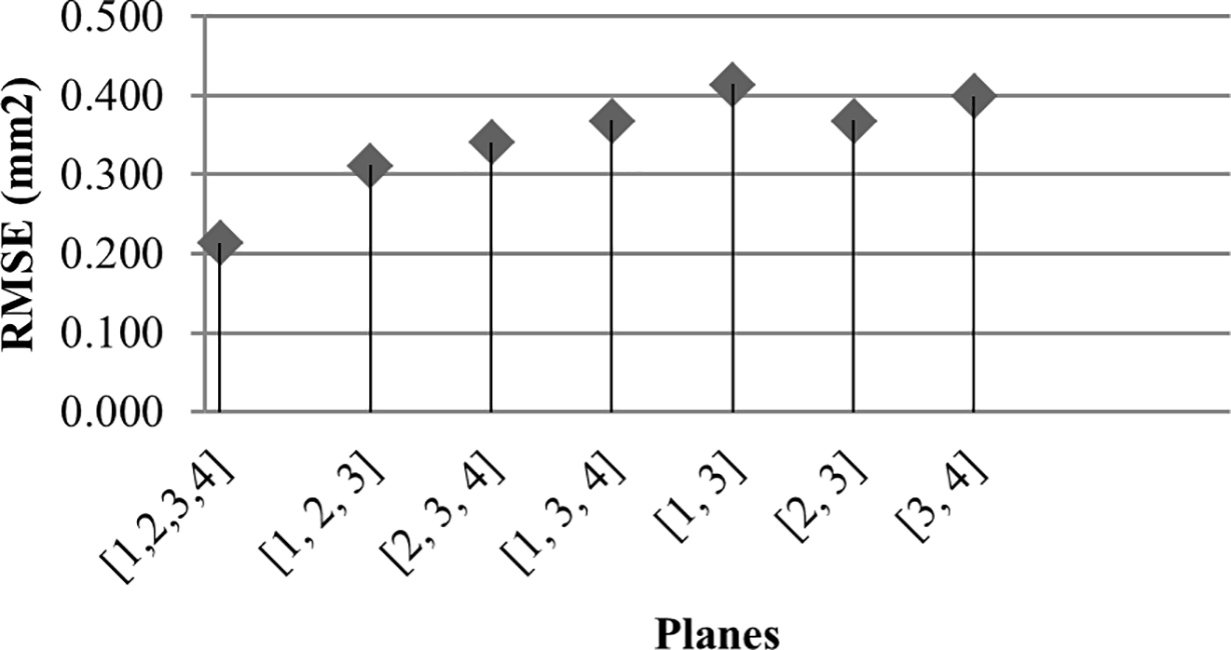
RMSE with subsets of the projection planes. Patient 5, Table 2.

For a thorough comparison, paired luminal cross-sectional areas were analysed. When compared with circular approximation and elliptical fitting, the proposed method showed a closer representation of OCT luminal shapes and a higher accuracy in computed cross-sectional areas. The highest improvement of the proposed method over the circular and elliptical approaches was, respectively, about 40% (Patient 6), and about 70% (Patient 3). It was observed that for small angles between reconstructed diameter vectors, the ellipse fitting approach tends to underestimate cross-sectional areas and can result in a degenerate ellipse. It is important to underline that the discrepancy between the cross-sectional areas generally depends on the number of projections employed in the reconstruction, as well as on their mutual orientation and their orientation with respect to the arterial segment of interest. Fig 13 shows the RSM discrepancy between OCT and our approach (Patient 5), with varying number of employed angiographic planes in the reconstruction. The lowest RSM error of 0.213mm^2^ was observed when all the four planes were employed in the 3D reconstruction; this error increased above 0.4mm^2^ when subsets of three and, then, two planes only were employed. Similarly, we observed that the RSME increased to 2.534mm^2^ for Patient 1, when two out of the three acquired planes were employed.

We also explored whether the proposed method may reflect an FFR measurement, a widely used physiological estimate of lesion severity. This is of potential clinical relevance; however, to extend this to a clinical validation would require a clinical study powered adequately and specifically designed for that purpose. We suggest that the level of validation provided in the current manuscript provides a rationale and justification for such a study. The comparison of volume reduction due to a plaque with the FFR measurements showed a good agreement in establishing the clinical significance of a stenosis, except for one patient. This was the patient with the lowest FFR value, who showed a highly irregular luminal surface. Such disagreement might indicate that the plaque morphology has implication on the fluid dynamics and that for the same degree of stenosis (volume reduction due to a lesion) hemodynamic parameters might dependent on the plaque shape, as claimed by recent works [42]. Overall, these results seems to be in line with a recent clinical study that investigates the relationship between 2D quantities measured on ICA images and FFR measurement, showing that the 2D minimum diameter correlates well with FFR value in intermediate coronary lesions [43].

### Future work

The 3D model obtained in this paper is suitable for isogeometric analysis (IGA) of blood flow in the coronary arteries [44]. Also, the model intends to preserve the lumen morphology of a lesion, and recent work has shown the implications of it on the flow dynamics [42]. In the future, computational fluid dynamics approaches may supplement the 3D anatomical information obtained by ICA data by providing accurate estimation of FFR [44–46].

Moreover, NURBS properties (e.g. local support property) make the 3D model suitable for a co-registration problem. For instance, co-registration of the 3D coronary artery tree with corresponding 3D volume rendering of the epicardial surface (extracted from cardiac magnetic resonance images) may help relating location and status of a stenosis with area of myocardium at-risk [47].
